# The Effect of Food on the Pharmacokinetics of the Potassium‐Competitive Acid Blocker Vonoprazan

**DOI:** 10.1002/cpdd.1009

**Published:** 2021-08-24

**Authors:** Darcy J. Mulford, Eckhard Leifke, Mark Hibberd, Colin W. Howden

**Affiliations:** ^1^ Research and Development, Phathom Pharmaceuticals Buffalo Grove Illinois USA; ^2^ Division of Gastroenterology University of Tennessee Health Science Center Memphis Tennessee USA

**Keywords:** food effect, metabolites, pharmacodynamics, pharmacokinetics, vonoprazan

## Abstract

Herein, we report a food‐effect study of vonoprazan, an oral potassium‐competitive acid blocker. In a phase 1, randomized, open‐label, crossover study, healthy subjects received a single 20‐mg dose of vonoprazan either following an overnight fast or 30 minutes after a high‐fat breakfast. Plasma vonoprazan levels were determined at 0 hour and at 17 subsequent assessment points up to 48 hours after dosing. After a 5‐day washout, subjects received a second 20‐mg vonoprazan dose in the alternative fed/fasted state (identical process repeated). Twenty‐four subjects completed the study. Vonoprazan exposure was not meaningfully affected by food. Geometric mean ratios for maximum concentration, area under the concentration‐time curve from time 0 to 24 hours, and area under the plasma concentration–time curve extrapolated to infinity obtained under fed and fasting conditions were 1.05 (90% confidence interval, 0.98‐1.12), 1.13 (1.09‐1.18), and 1.15 (1.11‐1.19), respectively. Four subjects experienced 6 adverse events that were all mild and considered unrelated to the study drug. Vonoprazan can be administered without regard to food intake.

Gastroesophageal reflux disease is one of the most prevalent gastrointestinal disorders in Western countries.^1^ Although proton pump inhibitors (PPIs) have markedly improved the treatment of acid‐related disorders, areas of medical need remain.^2^, ^3^, ^4^, ^5^ For improved control of 24‐hour intragastric acidity, strategies other than PPI treatment have been investigated, including the use of potassium‐competitive acid blockers (P‐CABs).

Vonoprazan fumarate is an orally active P‐CAB under development in the United States and Europe for the treatment of erosive esophagitis and, in combination with antibiotics, *Helicobacter pylori* infection. It is already approved for use in several Asian and South American countries. Vonoprazan has a chemical structure that differs from other P‐CABs in that it does not contain an imidazopyridine ring structure, which had been associated with hepatotoxicity observed with earlier P‐CABs that are no longer under development.^6^, ^7^, ^8^, ^9^ In contrast to PPIs, vonoprazan is acid stable and does not require an enteric coating to protect from acid degradation in the stomach.^10^, ^11^ Furthermore, vonoprazan does not require acid activation to bind to the proton pump. Vonoprazan is rapidly absorbed following oral administration, with median time of peak plasma concentrations (t_max_) typically occurring within 2 hours after once‐daily dosing.^12^, ^13^ The rate of elimination from the plasma allows for once‐ or twice‐daily dosing, with mean elimination half‐life (t_1/2_) values of 7 to 8 hours. Vonoprazan exhibits time‐independent pharmacokinetics, with steady‐state concentrations achieved by day 3 or 4 with once‐daily dosing.^13^, ^14^ There is little to no accumulation in plasma after multiple doses; pharmacokinetic parameters at doses from 10 to 40 mg are similar on days 1 and 7. Unlike PPIs, vonoprazan is primarily metabolized by cytochrome P450 (CYP) 3A4 and to a lesser extent by CYP2B6, CYP2C19, and CYP2D6, resulting in pharmacologically inactive metabolites, designated M‐I, M‐II, and M‐III; an additional inactive metabolite M‐IV‐Sul is formed by sulfotransferase 2A1 and CYP2C9 (the structures of these metabolites have been published by Sugano^15^ and by Yamasaki et al^16^).^14^, ^15^, ^16^ Nonclinical studies have shown vonoprazan to be an effective inhibitor of gastric acid secretion with faster and longer duration of action than PPIs.^12^, ^17^, ^18^, ^19^, ^20^ Single‐dose data from clinical studies showed that vonoprazan at doses of ≥20 mg is well tolerated and has a rapid onset (<4 hours) and prolonged duration of action (24 hours).^21^

The evaluation of the effect of food on the pharmacokinetics of orally administered drug is critical to determine proper instructions on drug intake for patients. For most PPIs, for example, intake in a fasting state, 30 to 60 minutes before a meal is recommended,^22^, ^23^ given their reduced bioavailability and efficacy when given with or after food.^24^, ^25^

Herein, we report the pharmacokinetic and safety/tolerability results of a food‐effect study of single doses of vonoprazan in healthy subjects.

## Methods

### Study Design

This was a randomized, open‐label, 2×2 crossover, phase 1 study stratified by gender in healthy Caucasian male and female subjects. The study was conducted at a single site in Northern Ireland (MDS Pharma Services, Belfast, Northern Ireland). The study was approved by an independent ethics committee (Edinburgh Independent Ethics Committee, Edinburgh, Scotland). Written informed consent was obtained from all subjects before study initiation.

### Study Population

The enrolled study population included 12 men and 14 women aged 18 to 45 years, of Caucasian ethnicity, considered eligible on the basis of study inclusion and exclusion criteria. To be eligible, subjects must have been in good health and have had clinical laboratory evaluations (including clinical chemistry, hematology, and urinalysis) within the reference range for the testing laboratory, or only minor deviations that were deemed by the investigator to be clinically nonsignificant. Additional inclusion criteria included a body mass index between 18 and 30 kg/m^2^ and a willingness to provide written informed consent before any screening procedures; abstention from caffeine, alcohol, and strenuous exercise from 72 hours before dosing until the follow‐up call/visit; and use of adequate contraception from screening, throughout the study, and for 30 days following the last dose of study medication (applicable to female subjects of childbearing potential who were sexually active) or for 12 weeks after the follow‐up visit (applicable to male subjects with female partners, both of whom were required to use contraception). In addition to caffeine and alcohol, subjects who had received the following were excluded from participation: nicotine products 6 weeks before the first dose of study medication; antibiotics within 28 days prior to initial screening; prescription medication or St. John's wort, ginseng, kava, ginkgo biloba, melatonin, herbal or homeopathic preparations, or other nutriceuticals within 28 days before the first dose; over‐the‐counter medication including salicylates (except when approved on a case‐by‐case basis) or vitamin supplements within 14 days before the first dose; and grapefruit or grapefruit products, Seville oranges (sour) or products, or poppy seeds within 7 days before the first dose.

### Interventions and Assessments

Subjects were randomized to either the fed/fasted state or fasted/fed state sequence, with randomization stratified by sex. Eligible subjects received a single 20‐mg dose of vonoprazan (given as two 10‐mg tablets) either following an overnight fast or 30 minutes after a breakfast (≈900 calories, with 62% of the calories from fat, 23% from carbohydrate, and 14% from protein).

After a 5‐day washout period, subjects received a second 20‐mg dose of vonoprazan in the alternative fed/fasted state, and the identical process was performed. During dosing in the fasted state, subjects fasted for 10 hours before receiving the study drug. During dosing in the fed state, subjects fasted for 10 hours and then consumed the breakfast, which was started 30 minutes before dosing and eaten in ≤30 minutes. Vonoprazan was taken with 240 mL of water, and subjects refrained from eating for 4 hours after dosing. Water (200 mL) was given to all subjects at 2 and 4 hours after dosing. All administrations of vonoprazan were taken under supervision.

Plasma concentrations of vonoprazan were determined using a liquid chromatography–tandem mass spectrometry assay with a validated concentration range of 0.1 to 100 ng/mL. A total of 864 samples were analyzed in 13 reported batches. The bioanalytical method utilized protein precipitation and extraction of plasma samples with acetonitrile‐formic acid (100:0.2, v/v) before analysis by liquid chromatography–tandem mass spectrometry. The liquid chromatograph–tandem mass spectrometer system consisted of an Acquity ultra‐performance liquid chromatography system (Waters Corp., Milford, Massachusetts) and an API5000 triple quadruple mass spectrometer (AB Sciex, Framingham, Massachusetts) with a turbo ion spray interface. The high‐performance liquid chromatography column was an Acquity UPLC BEH C18 (2.1 mm I.D., 100 mm, particle size 1.7 μm; Waters Corp.) maintained at 40^◦^C. The isocratic mobile phase consisted of acetonitrile/20 mmol/L ammonium formate (pH 3) (32:68, v/v). The flow rate of the mobile phase was 0.4 mL/min, and the total run time was 5.0 minutes.^26^ The calibration curve included 9 concentrations within the validation range, and the accuracy and precision of the method was assessed with quality control samples at concentrations of 0.1, 0.25, 5, and 80 ng/mL. Precision values, based upon relative standard deviation (RSD%) of quality control samples, were ≤4.8%. For incurred sample reproducibility, a total of 48 samples were reanalyzed. Greater than two‐thirds of the repeat results and original results were within 20% of each other, which was within the acceptance criteria. The accuracy values, based on the calibration standards across the range, were between 97.9% and 103.0%. The purity of the internal standard of vonoprazan was 100%. Overall, the method was determined to be reliable on the basis of validation results for accuracy, precision, specificity, linearity, and reproducibility.

Pharmacokinetic assessments were based on blood samples for plasma vonoprazan levels that were collected at 0 hours and at 5 minutes and 0.25, 0.5, 0.75, 1, 1.5, 2, 4, 6, 8, 10, 12, 16, 24, 30, 36, and 48 hours after dosing. The 24‐ and 48‐hour samples were collected before breakfast. Safety variables included physical examination findings; vital signs; electrocardiograms; clinical laboratory evaluations including hematology, chemistry, and urinalysis; and treatment‐emergent adverse events (AEs).

### Statistical Analysis

This crossover study was calculated to have at least 90% power for area under the plasma concentration–time curve (AUC) and maximum concentration (C_max_) to reject the null hypothesis that the fed subjects and fasted subjects were not equivalent (based on equivalence of 80%‐125% for 12 subjects per sequence group [total sample size, N = 24]), in favor of the alternative hypothesis that dietary states were equivalent. Assumptions included similar variability in exposure in this study compared with prior single‐dose pharmacokinetic data for vonoprazan,^21^ with expected ratios of means of 1.0 for both AUC and C_max_, standard deviation on a log*_e_* scale of 0.272, analysis in the natural log scale using *t*‐tests for differences in means, and performance of each *t*‐test at the 5% level.

The pharmacokinetic analysis set comprised all subjects who received vonoprazan and who had sufficient plasma concentration data to calculate at least 1 pharmacokinetic parameter, used for all pharmacokinetic summaries and statistical analyses. Descriptive statistics were used to summarize plasma pharmacokinetic parameters of vonoprazan at each scheduled time point by dietary state. Linear and semilogarithmic plots of the mean and individual plasma concentrations were generated. Pharmacokinetic parameters were derived using noncompartmental analysis methods, using the concentration‐time data for all evaluable subjects. Actual sampling times, rather than scheduled sampling times, were used where applicable. All of the reported pharmacokinetic parameters except t_max_ were log‐transformed before analysis to account for increasing variance with increasing means. For the food‐effect analyses, statistical comparisons of C_max_, AUC from time 0 to 24 hours (AUC_0‐24_), and AUC from time zero extrapolated to infinity (AUC_0‐∞_) were made to determine if a food‐effect difference was observed for vonoprazan and were conducted using an analysis of covariance model. All statistical analyses were performed using SAS version 9.1.

The safety analysis set comprised all enrolled subjects who received at least 1 dose of vonoprazan and was used for safety and baseline demographics summaries. All AEs were coded using the Medical Dictionary for Regulatory Activities version 11.1 and analyzed by descriptive statistics.

## Results

Twelve male and 14 female subjects were initially randomized. Two female subjects who were randomized to the fed/fasted sequence did not receive a dose of the study drug and did not complete the study; 24 subjects completed the study and are included in the pharmacokinetic and safety analysis sets. Baseline characteristics for the 24 evaluable subjects are shown in Table 1.

**Table 1:** Demographic and Baseline Characteristics

	N = 24
Mean age (SD), y	25.5 (6.09)
Sex, n (%)	
Male	12 (50)
Female	12 (50)
Race, n (%)	24 (100)
Caucasian	
Mean weight (SD), kg	72.6 (12.44)
Mean height (SD), cm	174.6 (10.78)

SD, standard deviation.

### Pharmacokinetic Results

**Figure 1:**
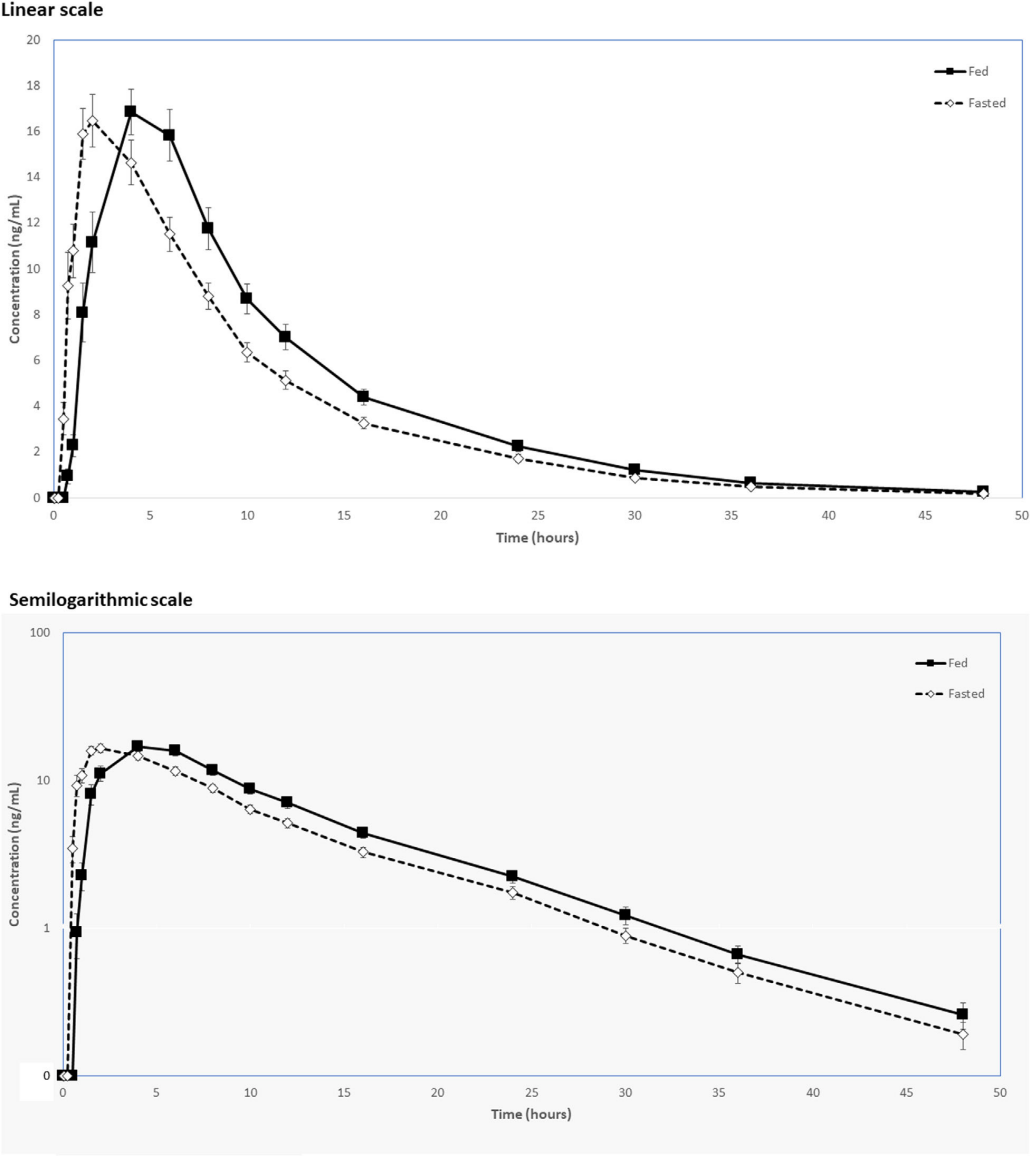
Mean vonoprazan plasma concentration versus time profiles following a single 20‐mg oral dose in fed and fasted conditions: (A) linear scale; (B) semilogarithmic scale.

Pharmacokinetic parameters of vonoprazan are summarized in Table 2. Overall, plasma concentrations (Figure 1) and exposure to vonoprazan were similar between the fed and fasted states. In the fed and fasted states, plasma concentrations of vonoprazan reached a peak by a median of 4.0 hours and 2.0 hours, respectively; thereafter, they gradually declined with a mean apparent terminal t_1/2_ of 6.9 hours in both states.

**Table 2:** Mean (SD) Pharmacokinetic Parameters for Vonoprazan Following a Single 20‐mg Oral Dose Under Fed and Fasted Conditions

	Fed (n = 24)	Fasted (n = 24)
C_max_, ng/mL	18.9 (5.12)	18.2 (5.76)
AUC_0‐24_, ng • h/mL	181.5 (52.84)	160.8 (49.50)
AUC_0‐∞_, ng • h/mL	206.1 (66.62)	179.3 (58.37)
t_max_, h, median (min, max)	4.0 (1.98, 6.02)	2.0 (0.75, 4.00)
t_1/2_, h	6.9 (1.21)	6.9 (1.45)

AUC_0‐∞,_ area under the plasma concentration–time curve from time 0 extrapolated to infinity; AUC_0‐24,_ area under the plasma concentration–time curve from time 0 to 24 hours; C_max_, maximum observed concentration; max, maximum; min, minimum; SD, standard deviation; t_max_, time at which the maximum observed concentration occurred; t_1/2_, terminal elimination half‐life.

The ratios of geometric means for C_max_, AUC_0‐24_, and AUC_0‐∞_ obtained under fed relative to fasting conditions were 1.05, 1.13, and 1.15, respectively. The exposure to vonoprazan was not meaningfully affected by food, based on the confidence interval from the analysis of variance being contained within the equivalence limits of 80% to 125% across the pharmacokinetic parameters tested (Table 3).

**Table 3:** Bioavailability of Vonoprazan Following a Single 20‐mg Oral Dose Administered Under Fed and Fasted Conditions (N = 24)

	Least‐Squares Means			
	Fed	Fasted	Fed/Fasted Ratio	90% CI
C_max_, ng/mL	18.2	17.3	1.05	0.98‐1.12
AUC_0‐24_, ng • h/mL	174.3	153.8	1.13	1.09‐1.18
AUC_0‐∞_ (ng • h/mL)	196.2	170.6	1.15	1.11‐1.19

AUC_0‐∞,_ area under the plasma concentration–time curve from time 0 extrapolated to infinity; AUC_0‐24_, area under the plasma concentration–time curve from time 0 to 24 hours; CI, confidence interval; C_max_, maximum observed concentration.

### Safety

Overall, vonoprazan was well tolerated. The incidence of AEs was similar during administration of vonoprazan in the fed state (2/24; 8.3%) and in the fasted state (3/24; 12.5%). The AEs were individual events of dizziness and rash in the fed state and dizziness, paresthesia, and nasal discomfort in the fasted state. All reported AEs were mild. None was considered possibly, probably, or definitely related to study treatment by the investigator. No subjects prematurely discontinued the study due to AEs.

No serious AEs and no deaths occurred during this study. However, after the end of the study period, 1 female subject experienced a serious AE of spontaneous miscarriage (early pregnancy loss), occurring ≈1 month after she had received vonoprazan. There were no clinically important changes in vital signs, 12‐lead electrocardiograms, or laboratory safety tests. In particular, there were no obvious treatment‐emergent changes in liver function tests. All physical examinations on admission and discharge were normal.

## Discussion

This phase 1 study explored the effects of food on the pharmacokinetics of single 20‐mg doses of vonoprazan. A comparison between the fed and fasted states indicates that exposure to vonoprazan is not materially affected by food. Vonoprazan was safe and well tolerated in healthy volunteers, following single 20‐mg doses given on 2 occasions. Our safety and tolerability findings are consistent with published data from other studies of vonoprazan in 212 healthy volunteers given daily doses of up to 40 mg for up to 7 days, with no safety signals.^13^, ^27^, ^28^, ^29^

A single‐dose food‐effect study of vonoprazan 20 mg in 12 healthy Japanese adult men showed that the AUC from time 0 to 48 hours (AUC_0‐48_) and C_max_ for vonoprazan were similar under fasted and fed conditions.^14^ In that study, the ratios between the fed/fasted states for unchanged vonoprazan were 1.08 for AUC_0‐48_ and 1.09 for C_max_, with 90% confidence intervals that were within the bioequivalence range (80%‐125%) for AUC_0‐48_ and just outside the upper bound of the range (1.26) for C_max_. Among subjects in the fasted state, C_max_ was 24.3 ng/mL, AUC_0‐∞_ was 225.3 ng·h/mL, and t_1/2_ was 7.7 hours; these were similar to those in the current study, which was conducted in exclusively Caucasian subjects. The median t_max_ was 4.0 hours in the fed state and 2.0 hours in the fasted state; these were also similar to the trend observed in the Japanese study, in which median t_max_ was 3.0 hours in the fed state and 1.5 hours in the fasted state.

Our findings are also consistent with repeated‐dose pharmacokinetic data for vonoprazan 20 mg, given once daily for 7 days after a ≥10‐hour fast in 2 separate studies; 1 was conducted in healthy male subjects in Japan and the other in Caucasian subjects in the United Kingdom.^13^ The t_max_ was 1.5 hours on both days 1 and 7 in the Japanese study, and 1.5 and 1.1 hours on days 1 and 7, respectively, in the UK study. While CYP2C19 genotyping was not performed in our study, prior observations from the Japanese repeated‐dose study^13^ as well as an earlier phase 1 study^21^ indicate that the pharmacokinetics of vonoprazan are not influenced by CYP2C19 polymorphisms, reflecting its predominant metabolism by CYP34A.^14^

The main limitation of this study is that subjects were administered only single doses of vonoprazan. Importantly, while other clinical studies have explored the pharmacokinetics of vonoprazan after multiple daily doses,^13^ they did not specifically assess the effect of food intake. Another limitation of this study is the absence of pharmacodynamic (intragastric pH) data, which may have provided additional insight into the effect of food intake on acid suppression following administration of vonoprazan.

In conclusion, vonoprazan can be administered without regard to food intake. In this study, it was well tolerated.

## Conflicts of Interest

D.J.M., E.L., and M.H. are employees of Phathom Pharmaceuticals. C.W.H. is a consultant for Alfasigma, Allakos, Clexio, Ironwood, Phathom Pharmaceuticals, and RedHill Biopharma.

## Funding

This study was sponsored by Takeda Global Research & Development Centre (Europe) Ltd.
